# Epidemiological and molecular characterization of dengue viruses imported into Guangzhou during 2009–2013

**DOI:** 10.1186/s40064-016-3257-3

**Published:** 2016-09-21

**Authors:** Yongxia Shi, Shufen Li, Xiaobo Li, Kui Zheng, Shuai Yuan, Jicheng Huang

**Affiliations:** Inspection and Quarantine Technology Center, Guangdong Entry-Exit Inspection and Quarantine Bureau, No. 13, Gangwan Road, Huangpu District, Guangzhou, 510700 Guangdong China

**Keywords:** Dengue virus, Epidemiological characterization, Imported case, Guangzhou

## Abstract

**Background:**

Dengue virus causes one of the most significant infectious diseases in tropical and subtropical regions, notable number of which is imported into China every year.

**Results:**

In this study, the molecular epidemiologic and phylogenetic analyses of dengue cases imported into Guangzhou in South China during 2009–2013 were conducted. During that period, 46 imported dengue cases were identified, including four serotypes. Most of the dengue patients were travelling from Southeast Asia, South Asia and Africa. The envelope (E) genes of 20 imported dengue viruses introduced from 13 countries and regions, were sequenced and used for phylogenetic analyses. The results indicated that the DENV-1 genotype I and DENV-2 Asian genotype I were the most predominant DENV strains, which were circulating in Southeast Asia and imported into South China. In addition, the new introduction of DENV-3 genotype III from West Asia was observed.

**Conclusions:**

This study provided an overview on the genetic diversity of DENV strains imported into South China, and also gave information about the geographic distribution, dynamic transmission and molecular evolution of epidemic DENV strains.

## Background

Dengue is one of the most prevalent mosquito-borne infectious diseases in tropical and subtropical regions, the incidence of which has been increasing considerably over the last decades (Mackey and Liang 2012). According to the statistics of World Health Organization (WHO), about 50–100 million dengue infections cases occurred every year in over 100 countries and regions, with severe infections mainly increased in Southeast Asian, Africa, South America and Western Pacific countries (Guzman et al. 2010; Arima et al. 2013; Bhatt et al. 2013).

Dengue virus (DENV), a member of genus *Flavivirus* in the family *Flaviviridae*, is the etiological agent of dengue, which is transmitted by *Aedes aegypti* and *Aedes albopictus* (Calisher et al. 1989). DENV is an envelope virus with a single-stranded positive sense RNA genome, which only contains one open reading frame encoding three structural proteins (including capsid protein, premembrane/membrane protein and envelope protein) and seven nonstructural proteins (NS1, NS2A, NS2B, NS3, NS4A, NS4B and NS5) (Halstead 2007). There are four antigenically distinct serotypes of DENV (DENV-1 to 4) (OhAinle et al. 2011), which cause diseases of dengue fever (DF), dengue hemorrhagic fever (DHF) and dengue shock syndrome (DSS) (Martina et al. 2009). Studies on the surveillance of imported dengue cases could provide information for the geographic distribution and dynamic transmission of DENV strains, which will benefit the prevention and control of dengue.

With the growth of global travel, the expansion and transmission of DENV strains into different regions of the world has been reported (Teichmann et al. 1999; Dorji et al. 2009; Shu et al. 2009). The Southeast Asia has been considered as a high-risk area (Shepard et al. 2013), since most dengue outbreaks occurred there, and the chance of acquiring dengue is high when traveling in these places (Chew et al. 2015; Zangmo et al. 2015). The provinces in the south of China share borders with several countries in Southeast Asia and the communication between China and these countries are more and more frequent, leading to the increasing importations of dengue into China. Guangzhou, a large city in South China, has the highest incidence of dengue in recent years. Dengue infections were reported in Guangzhou every year with the identification of four serotypes. By analyzing current circulation, epidemiological and molecular characterization of DENV strains imported into Guangzhou during 2009–2013, our study identified high-risk areas and epidemic seasons, providing information for the prevention and control of dengue.

## Methods

### Sample collection

Acute-phase blood samples were collected from different ports at Guangzhou in 2009–2013 under approval of patients. The suspected dengue cases were defined as patient had fever with two or more of the following symptoms: headache, arthralgia, conjunctival congestion, myalgia, rash, and chills. And the serum samples of suspected dengue cases were sent to the health and quarantine lab of Guangdong inspection and quarantine technology center with the consent of patients to confirm the infection of DENV and to conduct the subsequent study.

### Laboratory diagnosis

The DENV infection was defined as the detection of DENV RNA by real-time RT-PCR, using Ag-Path-ID™ Onestep RT-PCR Kit (ABI, USA). Real-time RT-PCR was performed with primers and probe listed in Table 1. The differentiation of DENV serotypes in the positive serum samples was conducted by using the detection kits of DENV I–IV (Liferiver, China).

**Table 1:** Primers/probe for detection of DENV and serotype-specific primers for amplification of E gene

Primers/probe	Sequences (5′–3′)
DENf	GCATATTGACGCTGGGARAGAC
DENr	GCGTTCTGTGCCTGGAWTGATG
DENp	FAM−CA+GAGA+TCC+TGC+TGTCTC−BHQ1
Den1-Ef	ATGCGATGCGTGGGAATAGG
Den1-Er	CGCCTGAACCATGACTCCTAGG
Den2-Ef	GCCTGCAGCTTCAATGACAATGCGTTG
Den2-Er	GCTCTAGACGGCCTGCACCATAAC
Den3-Ef	TGGGTTGACGTGGTGCTTGAGCACGG
Den3-Er	ATGGCTGTTGCCAATCTTTTTGGGGA
Den4-Ef	TCCAGCGAACTGTCTTCTTTGT
Den4-Er	AACCCGTGTCTGCTTGAACTGTG

### Viral RNA extraction, RT-PCR and envelope gene sequencing

Viral RNA was extracted from 140 µl of acute-phase serum by using QIAamp Viral RNA kit (QIAGEN, Germany) according to manufacturer’s instruction. The serotype-specific primers targeting the DENV envelope (E) gene listed in Table 1 were used in the amplification and sequencing of E gene. The amplification of E gene was conducted with QIAGEN OneStep RT-PCR Kit (QIAGEN, Germany) following the steps of 50 °C for 30 min, 95 °C for 15 min, and 30 cycles of 94 °C for 30 s, 50 °C for 30 s, 72 °C for 30 s; and a final extension at 72 °C for 7 min. The PCR products were purified and submitted to sequencing. All the sequences of envelope gene were submitted to GenBank under accession numbers: KX270802–KX270821.

### Phylogenetic analysis

A total of 20 DENV E gene were analyzed along with worldwide reference sequences of different genotypes,which are available on GenBank. The nucleotide sequences of DENV E genes were aligned, edited, and phylogenetically analyzed by using MEGA version 5.2. Phylogenetic trees were constructed by neighbor-joining method, with bootstrap value obtained from 1000 replicates. Sequences of D2/NewGuinea/NGC/1944 (M29095) and D3/Thailand/MK-315/1987 (L11442) were used as out-group to root the trees of DENV I–IV serotypes.

## Results

### Dengue virus imported into Guangzhou during 2009–2013

**Fig. 1:**
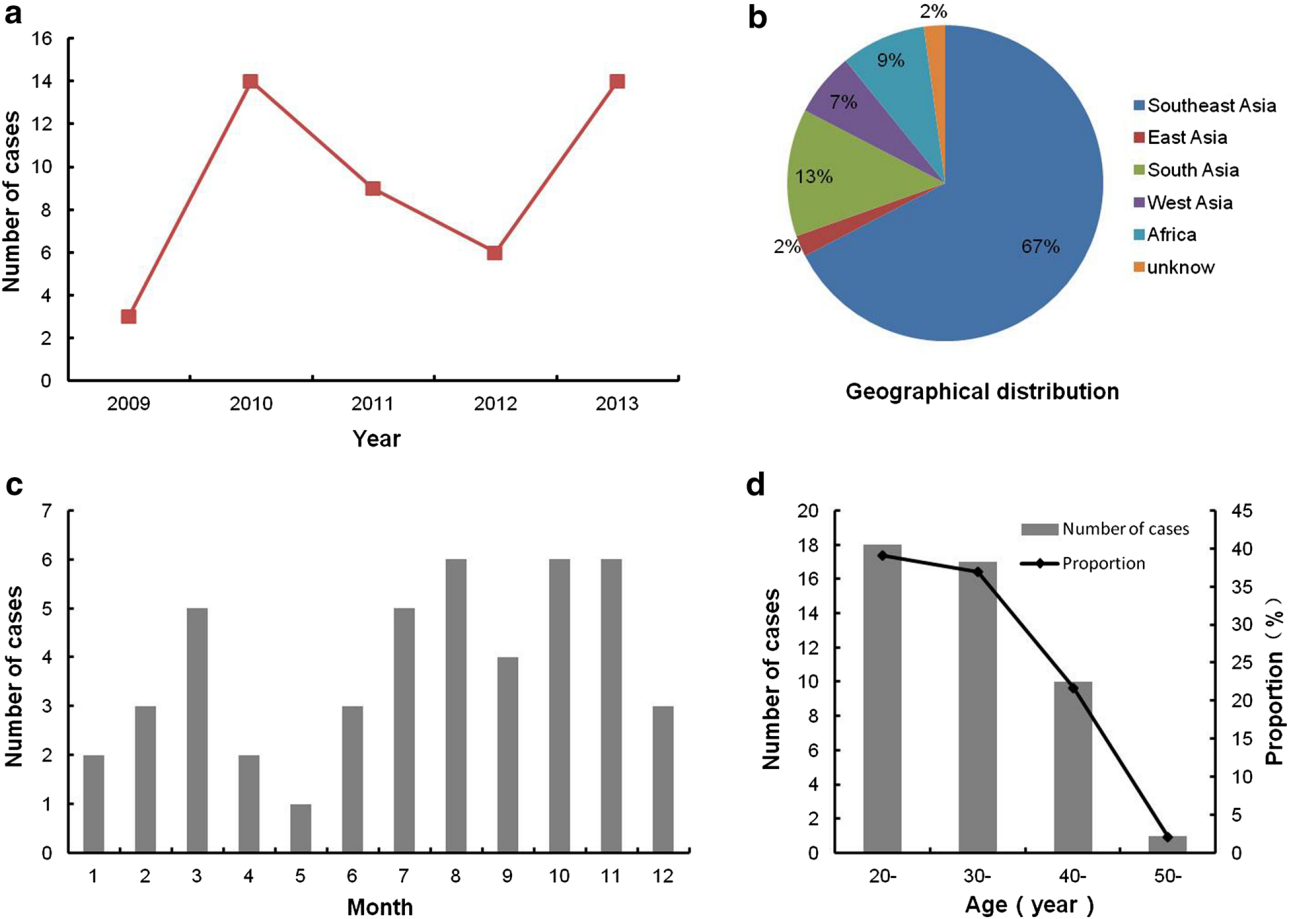
Summary of dengue virus imported into Guangzhou during 2009–2013. **a** The distribution of imported cases over years, data was collected during 2009–2013. **b** The geographical distribution of imported cases. **c** The temporal pattern of imported cases. **d** The age distribution of infected patients. Number of patients was indicated with the *bar chart*. The proportion of patients at different age phases to the total number was shown with the *line chart*

A total of 46 imported dengue cases were identified in Guangzhou during 2009–2013, all of which were detected by fever screening and diagnosed by real-time RT-PCR. According to the statistical result, number of imported case in the year of 2010 and 2013 was higher than other years (Fig. 1a). Most of the patients (31/46, 67.4 %) were infected in Southeast Asian countries, including Indonesia, Vietnam, Thailand, Philippines, Cambodia, Malaysia and Singapore (Fig. 1b). Patients from African regions were also identified. Other importing countries distributed in South Asia, West Asia and East Asia. The result indicated that Southeast Asia was the most important source of dengue cases imported into Guangzhou. The analysis of temporal pattern of imported cases indicated that positive samples could be detected through out of a year with a sporadic distribution pattern (Fig. 1c). Among all the cases, 40 (86.96 %) occurred in males and 6 (13.04 %) occurred in females. The ages of patients ranging from 20 to 50 (the median age was 30), and adults between 20 and 40 years old had the highest incidence (Fig. 1d).

### Serotype and genotype distributions of imported DENV

20 Positive imported cases from 13 countries were selected to conduct the serotype differentiation. The results indicated that 7, 10, 2 and 1 cases were infected with DENV-1, DENV-2, DENV-3, and DENV-4 strains, respectively. The genetic relationship of these DENV strains was determined by analyzing the E gene sequences of the viruses. The genotypes of DENV were differentiated as described previously. A summary of the serotype and genotype distributions of imported DENV during 2009–2013 was presented in Table 2.

**Table 2:** List of DENV strains used in phylogenetic analysis

Strains	Serotype	Genotype	Year of isolation	Country origin	GenBank No.
B09H004096	DENV-1	I	2009	Vietnam	KX270805
01011100000237	DENV-1	I	2011	Philippines	KX270807
01011200000861	DENV-1	I	2012	Cambodia	KX270803
01011200000371	DENV-1	III	2012	Africa	KX270802
0101130000079	DENV-1	III	2013	Indonesia	KX270806
01011300000201	DENV-1	IV	2013	Malaysia	KX270804
01011100000465	DENV-1	V	2011	Pakistan	KX270808
B10H001114	DENV-2	Asia I	2010	Thailand	KX270817
B10H001132	DENV-2	Asia I	2010	Thailand	KX270818
01011100000441	DENV-2	Asia I	2011	Cambodia	KX270810
01011300000154	DENV-2	Asia I	2013	Singapore	KX270813
B10H001113	DENV-2	Cosmoplitan	2010	Bangladesh	KX270816
B10H001104	DENV-2	Cosmoplitan	2010	Vietnam	KX270815
01011100000388	DENV-2	Cosmoplitan	2011	India	KX270809
01011300000191	DENV-2	Cosmoplitan	2011	Turkey	KX270814
01011200000481	DENV-2	Cosmoplitan	2012	Singapore	KX270811
01011200000487	DENV-2	Cosmoplitan	2012	Hong Kong	KX270812
01011300000094	DENV-3	I	2013	Thailand	KX270819
01011100000191	DENV-3	III	2013	Turkey	KX270820
B10H001124	DENV-4	II	2010	Philippines	KX270821

### Phylogenetic analysis of DENV-1 strains

**Fig. 2:**
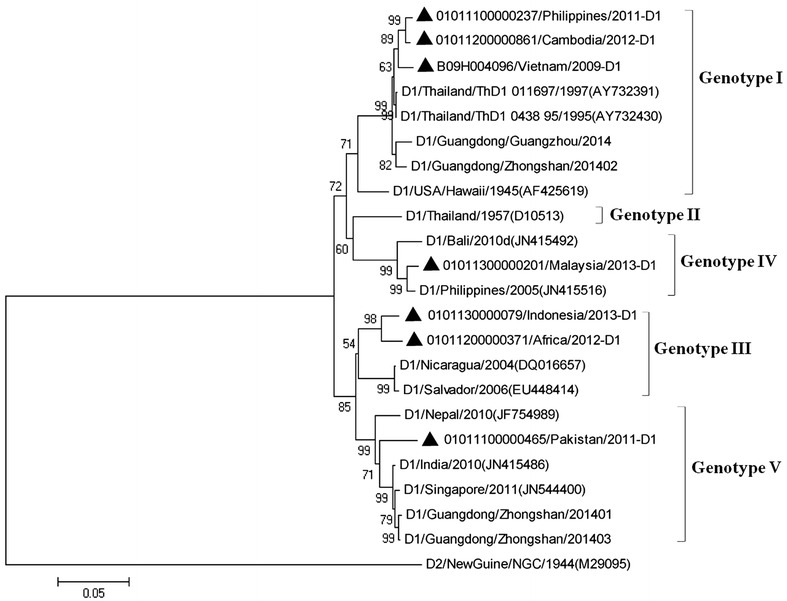
Phylogenetic tree of dengue virus type 1 (DENV-1). The phylogenetic tree is based on the complete E gene sequences of DENV-1 serotype, and constructed by using the neighbor-joining method (MEGA 5.2) with bootstrap 1000 replications. The sequences were named according to strain/country/year/serotype of collected viruses. GenBank accession numbers of the reference sequences are shown in the *parentheses*. GenBank accession No. of imported strains were listed in Table 2. The imported cases are designated in *solid triangle*

The phylogenetic tree of DENV-1 strains was generated using E gene sequences of 7 DENV-1 strains and 11 reference strains belonged to corresponding genotypes (Fig. 2). The results revealed that genotypes I was the most predominant genotype of DENV-1 strains, which were imported from Southeast Asian countries, including Philippines, Cambodia and Vietnam. None of 7 DENV-1 strains belonged to genotype II. While two of them were classified into genotype III, one was imported from Indonesia and the other from Africa. Both of the genotype III strains were closely related to the virus from Latin America. It is notable that the geographical distributions of genotype III viruses are diverse, including Asia, Latin America and Africa. Genotype IV and V contains one strain from Malaysia and Pakistan, respectively.

### Phylogenetic analysis of DENV-2 strains

**Fig. 3:**
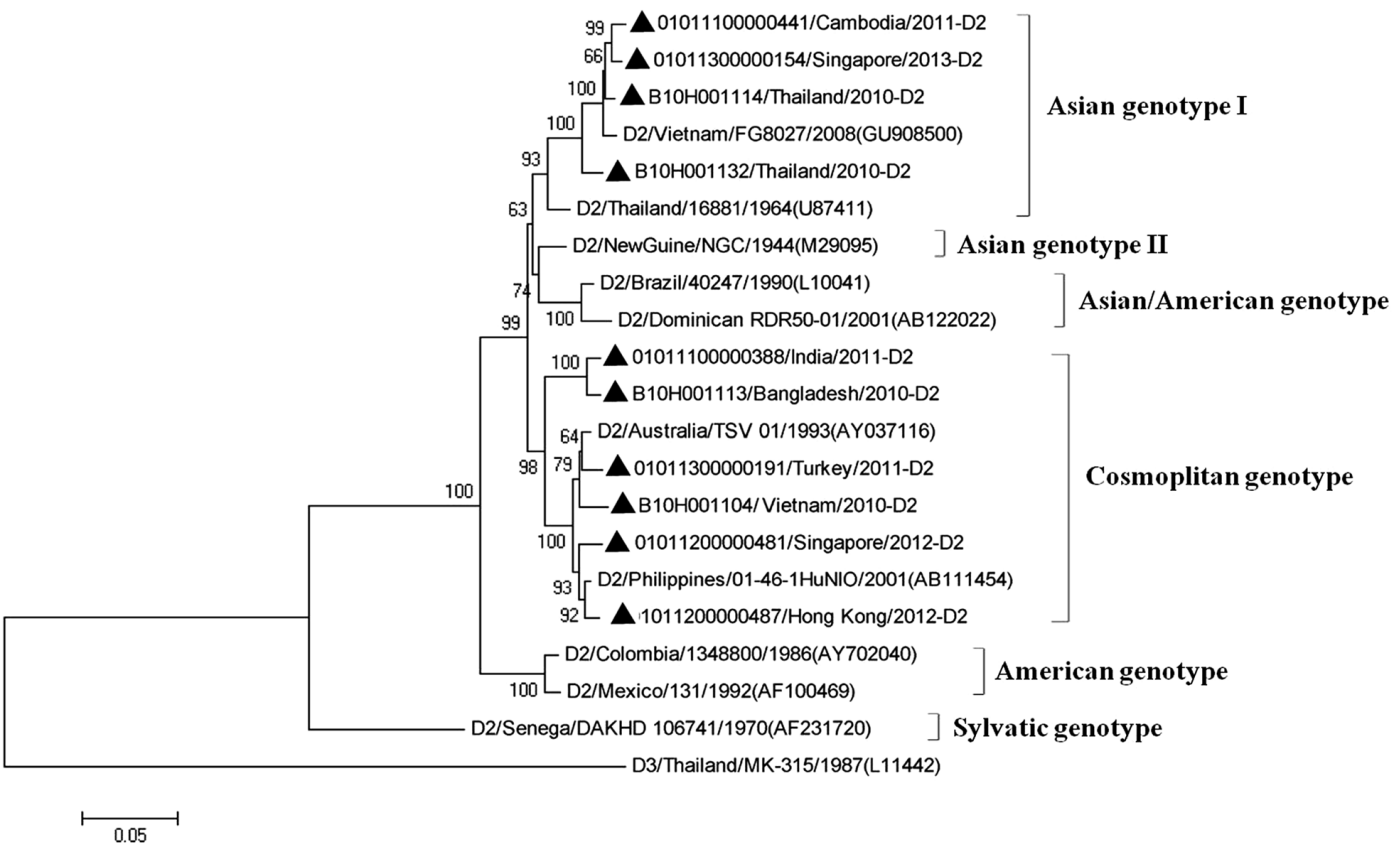
Phylogenetic tree of DENV-2. The phylogenetic tree is based on the complete E-gene sequences of DENV-2 including 10 imported dengue cases during 2009–2013, and constructed by using the neighbor-joining method with bootstrap 1000 replications. The sequences were named according to strain/country/year/serotype of collected viruses. GenBank accession numbers of the reference sequences are shown in the *parentheses*. GenBank accession No. of imported strains were listed in Table 2. The imported cases are designated in *solid triangle*

The E gene sequences of ten DENV-2 strains were aligned with ten reference strains of six genotypes and used to construct phylogenetic tree (Fig. 3). The DENV-2 strains imported into South China during 2009–2013 were clustered into two genotypes, Asian genotype I and Cosmopolitan genotype. Among them, four virus strains clustered into Asian genotype I was from Southeast Asia, including Cambodia, Singapore and Thailand. The strains of Cosmopolitan genotype showed a wider geographical distribution, which were imported from Southeast Asia, South Asia and West Asia. The exporting countries and regions comprised India, Bangladesh, Turkey, Vietnam, Singapore and Hong Kong. No Asian genotype I, Asian/American genotype, American genotype and Sylvatic genotype were detected in imported cases during 2009–2013. Three strains of Asian genotype I were clustered into a clade with the viral strain from Vietnam. The strain of Cosmopolitan genotype from Turkey was closely related to Australia strain.

### Phylogenetic analysis of DENV-3 strains

**Fig. 4:**
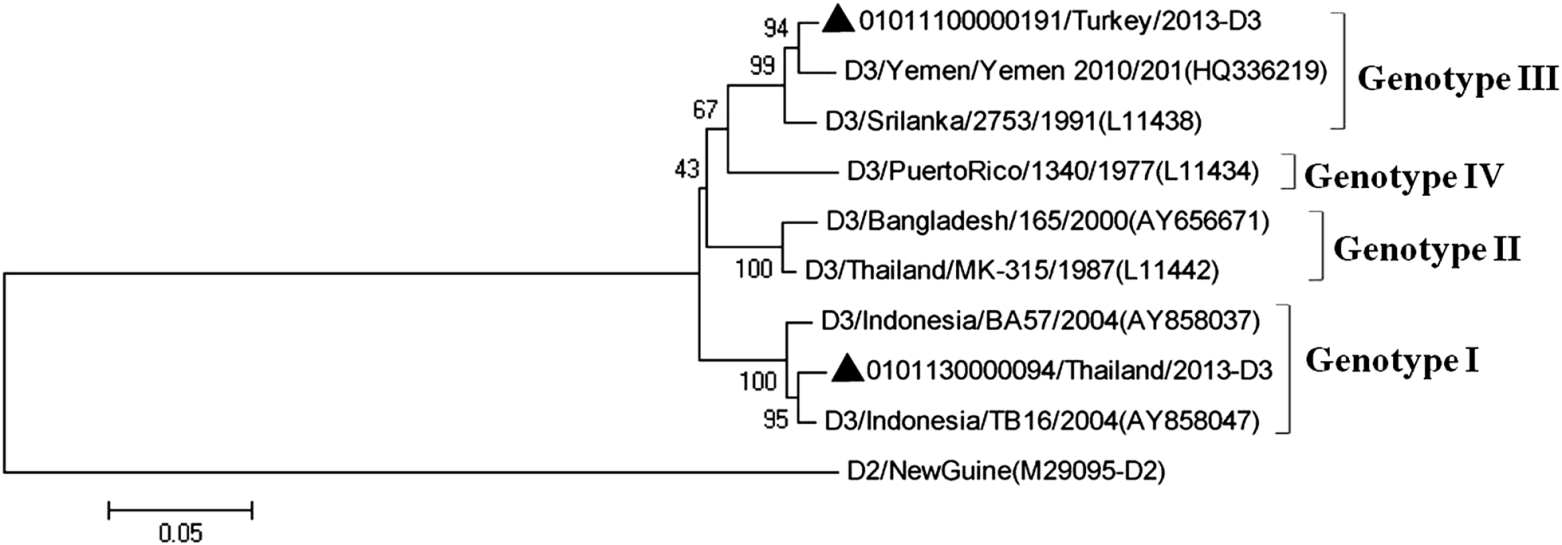
Phylogenetic tree of DENV-3. The phylogenetic tree is based on the complete E gene sequences of nine strains of DENV-3 including two imported cases, and constructed by using the neighbor-joining method with bootstrap 1000 replications. The sequences were named according to strain/country/year/serotype of collected viruses. GenBank accession numbers of the reference sequences are shown in the *parentheses*. GenBank accession No. of imported strains were listed in Table 2. The imported cases are designated in *solid triangle*

Two DENV-3 strains were determined in the imported cases, which belonged to genotype I and genotype III, respectively (Fig. 4). Genotype III Strain imported from Turkey was clustered into clade with Middle East source virus, while genotype I strains from Thailand was closely related to strains from Indonesia. No strain belonged to genotype II and genotype IV was detected in imported cases during 2009–2013.

### Phylogenetic analysis of DENV-4 strains

**Fig. 5:**
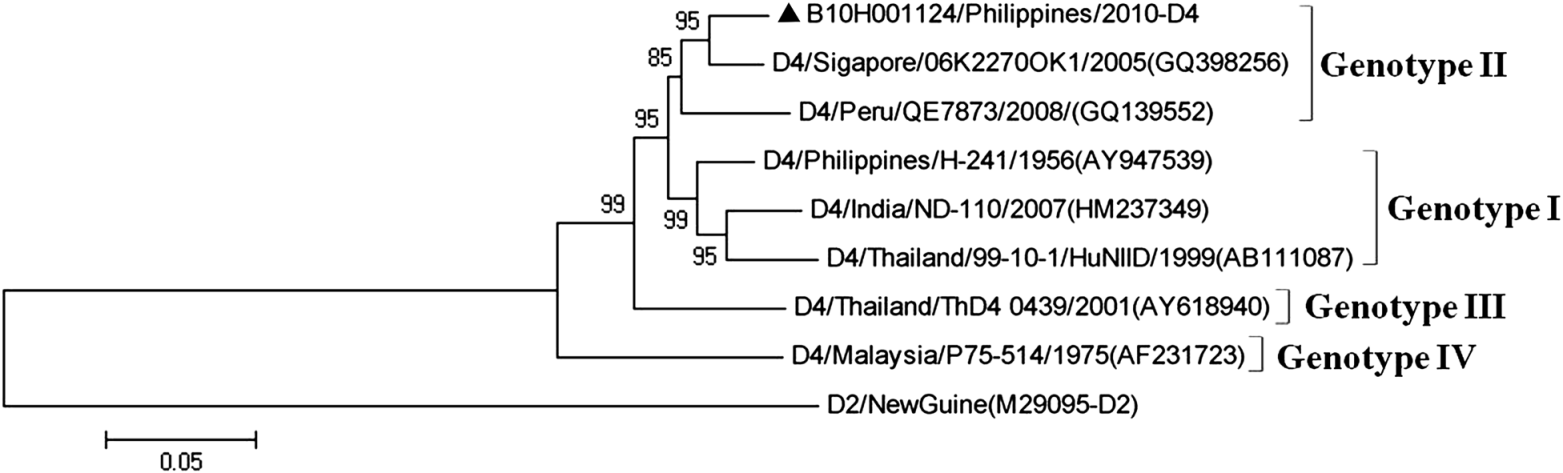
Phylogenetic tree of DENV-4. The phylogenetic tree is based on the complete E gene sequences of nine strains of DENV-4, and constructed by using the neighbor-joining method with bootstrap 1000 replications. The sequences were named according to strain/country/year/serotype of collected viruses. GenBank accession numbers of the reference sequences are shown in the *parentheses*. GenBank accession No. of imported strains were listed in Table 2. The imported cases are designated in *solid triangle*

According to our data, DENV-4 is the least frequent serotype circulating in Asia; only one strain was detected in the imported cases, which was from Philippines (Fig. 5). The result of Phylogenetic analysis indicated that Philippines strain belonged to genotype II, which was closely related to viruses from Singapore and Peru.

## Discussion

Dengue is one of the most significant infectious diseases in tropical and subtropical regions, in recent years, the communication among countries showed a rising trend due to the globalization, which caused an increased number of imported dengue cases worldwide. As Guangzhou is located in South China and close to Southeast Asian countries, it is in high risk of dengue virus importation. In this study, the introduction of DENV into Guangzhou during 2009–2013 was investigated. 46 serum samples were detected to be DENV positive; most of them (31/46, 67.4 %) were introduced from Southeast Asian countries, indicating that Southeast Asia was the high-risk area. The number of imported case in 2010 and 2013 was higher than other years, which was related to the outbreak of Dengue in Southeast Asian countries (Fig. 1). 20 of the imported cases were chosen for the serotype differentiation and molecular characterization. The results suggested that all of the four serotypes were introduced to South China, with DENV-1 and DENV-2 as the most predominant serotypes (Table 2). The number of dengue strains introduced from South and West Asia showed an increased trend.

Further genotype analysis indicated that DENV-1 genotype I and DENV-2 Asian genotype I were the most predominant DENV strains circulating in Southeast Asian countries, while DENV-2 Cosmoplitan genotype comprised the largest part of the imported strains (Table 2). The importation of DENV-2 Asian genotype II and Asian/American genotype from Southeast Asia was not identified, in consistent with previous reports (Huang et al. 2012). DENV-3 genotype III, which was recently considered as an emerging DENV genotype in Southeast Asian countries, was found to import into South China from West Asia (Table 2). In addition, one strain of DENV-4, belonged to genotype II, was introduced from Philippines (Table 2).

It has been reported that DENV-1 genotype I became the most predominant genotype circulating in Southeast Asia in 1980s, replacing the genotype III viruses. In this study, during 2009–2012, DENV-1 strains imported from Vietnam, Philippines and Cambodia, belonged to genotype I and clustered with two Thailand strains (ThD1 0438 95 and ThD1 011697) (Fig. 2). In 2012 and 2013, strains imported from Africa and Indonesia were clustered together with genotype III viruses from Central American countries (Fig. 2), including Nicaragua and Salvador, suggesting the exportation of DENV from this region. One case of genotype IV was imported from Malaysia in 2013 (Fig. 2), which year a large number of dengue infection was detected and reported in Malaysia (Chew et al. 2015). A genotype V strain was introduced from Pakistan in 2011 (Fig. 2), which is consistent with the fact that large epidemics began to emerge in Pakistan since 2011 (Wesolowski et al. 2015). A large outbreak of dengue occurred in Guangzhou in 2014, with DENV-1 as the most predominant serotype, which was caused by indigenous cases and imported cases from Southeast Asian countries (Huang et al. 2016). Phylogenetic characterization revealed that the strains belonged to genotypes I, IV and V of DENV-1 (Huang et al. 2016; Sun et al. 2016). During 2009–2013, DENV-1 genotypes I, IV and V were detected to be the most predominant genotypes introduced to Guangzhou (Fig. 2), which might provide a hint to the dengue epidemic in Guangzhou in 2014.

The result of phylogenetic analysis of envelope protein gene of DENV indicated that Asian genotype I of DENV-2 was the predominant DENV-2 lineage in Southeast Asia during 2009–2013 (Fig. 3), as Asian genotype I strains was imported from Thailand, Cambodia and Singapore. This was consistent with the report that Asian genotype I emerged in Southeast Asia in recent years (Vu et al. 2010). A notable shift from the Asian genotype II and Asian/American genotype to Asian genotype I and Cosmopolitan genotype was found, since all of the DENV-2 strains were clustered into Asian genotype I and Cosmopolitan genotype (Fig. 3). Genotypes including Asian genotype II, Asian/American genotype, American genotype and Sylvatic genotype were not founded in imported dengue cases during the study period, suggesting a low prevalence rate of these genotypes in the regions communicating with South China.

The introduction of DENV-3 into Guangzhou was observed in 2013 (Fig. 4), one strain imported from Thailand was clustered with genotype I strains from Indonesia. The genotype III strain introduced from Turkey was clustered with strains from Yemen and Sri Lanka. The genotype III used to distribute in East Africa, Latin America and South Asia. Recent reports showed that the strains of DENV-3 genotype III were imported into Southeast Asia, South China and Taiwan (Sun et al. 2011; Huang et al. 2012), indicating that the geographic distribution of DENV-3 genotype III had expanded to Southeast Asian regions. The incidence rate of DENV-4 virus infection was low in Southeast Asia, in this study, only one strain imported from Philippine was clustered with strains belonged to DENV-4 genotype II (Fig. 5).

In order to prevent the outbreaks of DENV infection in China, more education should be provided for people traveling or working at the epidemic areas, especially Southeast Asia countries, to remind them of personal protection. At the meanwhile, the local hygienic authorities should reinforce the surveillance work towards people and goods from epidemic areas to discover and deal with the suspicious patients and vectors in a timely manner. DENV is a mosquito-borne virus, which could be transmitted by *A. aegypti* and *A. albopictus.* The *Aedes* mosquitoes are wildly distributed in various provinces in Mainland China, thus, it is crucial to take measures to control the local mosquitoes for the prevention of further spread.

## Conclusions

In this study, we conducted the molecular epidemiologic and phylogenetic analysis of dengue strains imported into Guangzhou in South China during 2009–2013. The results provided an overview on the genetic diversity of DENV stains imported into South China, which also gave information about the geographic distribution, dynamic transmission and molecular evolution of DENV stains in epidemic regions, especially in Southeast Asia. In addition, the continuous surveillance on the imported dengue case could benefit the prevention of further dengue outbreaks in Guangzhou.
